# Predictive risk factors of venous thromboembolism (VTE) associated with peripherally inserted central catheters (PICC) in ambulant solid cancer patients: retrospective single Centre cohort study

**DOI:** 10.1186/s12959-019-0191-y

**Published:** 2019-01-25

**Authors:** Osamah Al-Asadi, Manar Almusarhed, Hany Eldeeb

**Affiliations:** 1 Department of Oncology, Milton Keynes University Hospital, Milton Keynes, UK; 2 School of Medicine, University of Buckingham, Buckingham, UK; 3 College of medicine, Al-Mustansiriyah University, Baghdad, Iraq; 4 College of medicine, Babylon University, Babylon, Iraq

## Abstract

**Aims:**

Peripherally inserted central catheters(PICC) lines are becoming increasingly popular in solid cancer patients for the administration of chemotherapy. This study aims looking at the incidence of PICC line related and distant thromboembolism associated with these catheters and exploring risk factors.

**Methods:**

Records were reviewed for 158 patients who underwent PICC line insertion over the two years period in the medical oncology unit, Milton Keynes University Hospital. The Incidence PICC line related Deep Venous Thrombosis (DVT) which is defined as upper extremity DVT at the site of PICC line insertion was documented after checking reports of ultrasound Doppler of all symptomatic patients to confirm the presence of thrombo-embolism and Computed Tomography(CT)scan or Computed Tomography Pulmonary Angiography (CTPA) to confirm the presence Pulmonary Embolism(PE).

**Results:**

23(13%) symptomatic patients with confirmed diagnosis by ultrasound Doppler were found to have PICC line related DVT and similar number of patients developed distant VTE, namely PE and lower limbs DVT. Average time to thrombo-embolism from the insertion of PICC line was 13 days and 51 days in distant VTE. Statistically significant results have been identified in the term of risk factors leading to VTE events during the period of PICC line insertion.

**Conclusions:**

VTE is a common complication in medical oncology patients who underwent insertion PICC line insertion for chemotherapy. Risk of distant VTE is high as well as the PICC line related DVT and the risk of the PICC line related DVT is higher in the first two weeks after PICC insertion. We concluded that high BMI,high PLTs count and Fluropyrimidine containing chemotherapy are all significant risk factors for VTE events recorded while smoking and high BMI are significantly contributing to the high rate of the PICC line related DVT.

## Introduction

The use of PICC line has grown significantly in hospitalized patients in comparison with central venous catheters reflecting their clinical advantages besides avoiding iatrogenic complications frequently associated with central venous catheters [1, 2]. As the PICC line terminated in central veins, they can be used for the infusion of chemotherapy in an outpatient setting for cancer patients [3, 4].However, accumulating evidence suggests that PICC lines are associated with important complications, including upper-extremity DVT,PE, loss of Intravenous access and post thrombotic syndrome [5–11]. The PICC line related DVT in patients with cancer leads to increasing morbidity and mortality [12]. Unfortunately, no much work has been conducted to look at the incidence and risk factors for VTE in cancer patients following the insertion of PICCs as published researches had focused on all central venous catheters of which just 15% were PICCs [13]. The incidence of PICC related VTE varies widely in different studies, with the symptomatic VTE in this scenario is reported to be varying around 6% to up to 18% and in few studies, it was around 25% [1, 3] while the rate of asymptomatic thrombosis has been reported to be up to 35–71.9% [14–18]. Many of these studies use a retrospective design and the actual rate of PICC related DVT is still not well defined. It is very important to further explore the incidence and risk factors for PICC line related venous thrombosis. In our study we analyse our experience with PICC insertion in the ambulatory solid cancer patient’s population and measure the incidence of local, distant VTE events and relative risk factors.

### Aims

To assess the incidence of PICC related VTE in solid cancer patients in the ambulatory setting who underwent PICC line insertion in Macmillan Unit, Milton Keynes university hospital in two years. We also aim to identify any patient related or catheter related risk factors. Also, we measure the incidence of distant VTEs like PE and lower-limbs DVT that developed during the period of PICC line insertion.

## Method

All patients with solid cancers who received chemotherapy through the PICC line for the period January 2016 to December 2017 were included. Electronic patient records were reviewed for their age, gender, history of antiplatelet or anticoagulation, pervious risk factor for VTE, chemotherapy type, smoking, Body Mass Index(BMI), White Blood Cell(WBC) count, Platelet (PLT) count, prior PICC line insertion in the last 6 months, pathological diagnosis and treatment setting such as early (neoadjuvant and adjuvant) vs palliative setting. The Incidence of PICC line related DVT which is defined as upper extremity DVT at the site of PICC line insertion and distant VTE were confirmed on ultrasound Doppler in all symptomatic patients and CT scan or CTPA.

### Statistical analysis

Data analysis was made using SPSS software, version 22 (IBM Corp., Armonk, NY, USA). Descriptive statistics for continuous variables included mean, median and range. The number and proportions of the categorical data were used to characterize the demographics and baseline of the study population. A multivariate logistic regression model was conducted to examine the association between risk factor and development of VTE events. Multicollinearity was assessed using variance inflation factors and spearman correlations. In all analysis, the null hypothesis was rejected at 5% as a cut of the point for Significance (two-tailed) testing.

## Results

Total of 180 patients were retrospectively evaluated for PICC insertion. 22 patients have been excluded due to incomplete data.158 patients with completed data were found eligible for analysis. The PICC line related DVT was identified in 23 (13%) of symptomatic patients with confirmed diagnosis by ultrasound Doppler while distant VTE was identified in 23(13%) of patients in the cohort; two of them had the PICC line related DVT already. The distribution of distant VTE was 18(78%) patients as PE while the lower limbs DVT recorded in only 5(22%) patients. The mean age of the participants at the time of PICC insertion was 57 with a range of 27 to 80. 102 (64%) patients were female. The primary diagnosis was Colorectal cancer (*n* = 78, 49.4%), followed by breast cancer (*n* = 63, 39.9%) and pancreatic cancer (*n* = 9, 5.7%). Only 13 (8.2%) had prior PICC insertion in the last 6 months. Regarding the treatment intention, 96 (60.7%) were on palliative treatment, 41 (26%) were on adjuvant treatment, and 21 (13.3%) were on neoadjuvant. Smokers represent 18.9% (30 patients)of the cohort. The previous risk factor for VTE was found in 25 patients (15.9%) of the cohort. Twelve (7.6%) patients had a previous VTE event. Fluropyrimidine containing chemotherapy was given in 80% of the patients. The baseline characters are shown in the Table 1. After PICC insertion, the median time required for PICC line related DVT development was 13 days 95% CI (12, 32) and 51 days 95% CI (45, 77) in distant VTE.

**Table 1:** Baseline characteristics and patient demographic of the study cohort

Characteristic	All patients (*n* = 158)	PICC-related DVT (*n* = 23)	Distant VTE (*n* = 23)
Age (Mean)	57	52	62
Gender			
Male	56 (35.4%)	7 (12.5%)	9 (16%)
Female	102 (64.6%)	16 (15.7%)	14 (13.7%)
Primary cancer			
Breast	63 (39.9%)	14 (22.2%)	2 (3.1%)
Colorectal	78 (49.4%)	8 (10.2%)	17 (21.8%)
Pancreas	9 (5.7%)	1 (11.1%)	3 (33.3%)
Other	8 (5%)	0	1 (12.5%)
Treatment intention			
Neoadjuvant	21 (13.3%)	6 (28.6%)	0
Adjuvant	41 (26%)	8 (19.5%)	1 (2.4%)
Palliative	96 (60.7%)	9 (9.3%)	22 (23%)
History of antiplatelet or anticoagulation	14 (8.9%)	2 (14.3%)	2 (14.3%)
Previous VTE event	12 (7.6%)	2 (16.7%)	1 (8.3%)
Smoking	30 (18.9%)	7 (23.3%)	2 (6.6%)
Pervious risk factor for VTE	25 (15.9%)	2 (8%)	4 (16%)
Chemotherapy type			
Fluropyrimidine containing	127 (80.3%)	21 (16.5%)	21 (16.5%)
Fluropyrimidine non-containing	31 (19.7%)	2 (6.4%)	2 (6.4%)
Prior PICC line in last 6 months	13 (8.2%)	1 (7.7%)	4 (30.8%)

*PICC* peripherally inserted central catheter, PRDVT PICC related *DVT, VTE* vascular thromboembolism

Table 2 shows the logistic regression model that included all potential predictive factors for VTE events. This model indicated that smoking increased the risk of PICC related DVT by four.

**Table 2:** Logistic regression model of factors with potential predictive value

Parameter	PRDVT		Distant VTE		All VTE	
	Odds Ratio (95%CI)	*P* value	Odds Ratio (95%CI)	*P* value	Odds Ratio (95%CI)	*P* value
Age on insertion	0.97 (0.92, 1.02)	0.270	1.02 (0.97, 1.08)	0.393	1.00 (0.96, 1.04)	0.971
Female	0.29 (0.54, 1.59)	0.155	2.50 (0.75, 8.33)	0.135	1.81 (0.65, 5.04)	0.254
Primary cancer type						
Breast	N/A	N/A	1.18 (0.04, 31.75)	0.922	2.76 (0.169, 45.00)	0.746
Colorectal	N/A	N/A	0.84 (0.03, 26.54)	0.923	1.07 (0.09, 13.65)	0.959
Pancreas	N/A	N/A	0.82 (0.02, 42.66)	0.920	2.82 (0.137, 58)	0.502
Intention to treatment						
Adjuvant	1.16 (0.25, 5.42)	0.851	N/A	N/A	1.43 (0.33, 6.198)	0.629
Palliative	1.33 (0.12, 15.32)	0.187	N/A	N/A	4.62 (0.57, 37.56)	0.153
Ongoing antiplatelet or anticoagulation therapy	1.31 (0.21, 8.22)	0.777	2.91 (0.34, 24.76)	0.329	1.30 (0.30, 5.69)	0.730
Previous VTE event	1.28 (0.17, 9.77)	0.814	0.27 (0.02, 2.91)	0.277	0.77 (0.16, 3.61)	0.740
Smoking	3.90 (1.09, 13.98)	0.036*	0.29 (0.05, 1.66)	0.166	1.61 (0.59, 4.40)	0.351
Pervious risk factor for VTE	0.40 (0.07, 2.42)	0.315	0.66 (0.12, 3.36)	0.612	0.54 (0.15, 1.95)	0.348
Fluropyrimidine containing chemotherapy	5.87 (0.69, 50.22)	0.106	6.80 (0.21, 224.50)	0.283	10.06 (1.6, 63.05)	0.014*
BMI ≥ 30	1.11 (1.01, 1.23)	0.026*	1.12 (1.00, 1.23)	0.033*	1.13 (1.05, 1.22)	0.001*
WBC count	0.86 (0.69, 1.08)	0.202	1.08 (0.95, 1.23)	0.218	1.01 (0.91, 1.12)	0.865
Platelet count	1.01 (1.00, 1.01)	0.103	1.00 (1.00, 1.01)	0.245	1.00 (1.00, 1.01)	0.034*
Prior PICC line in last 6 months	0.74 (0.08, 7.14)	0.791	3.97 (0.80, 19.78)	0.092	2.16 (0.58, 8.03)	0.252

*DVT* deep vein thrombosis, *PICC* peripherally inserted central catheter, PRDVT*, PICC related DVT, VTE* vascular thromboembolism, *CI* confidence interval, WBC white blood cells, BMI body mass index, *N/A* not applicable

**Fig. 1:**
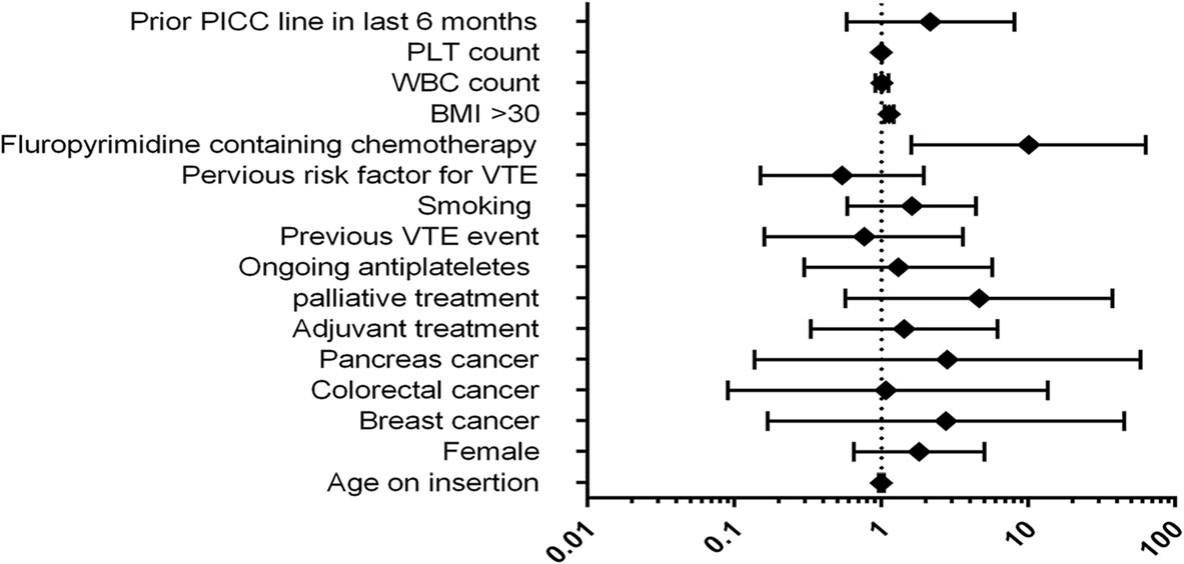
summary of odds ratios of all potential predictive factors

*= *P* < 0.05 statistically significant

fold (Odds Ratio [OR] 3.9, 95% Confidence Interval [CI] 1.01, 13.98). Also, Body mass index was a significant predictor (Odds Ratio [OR] 1.11, 95% Confidence Interval [CI] 1.01, 1.23). Regarding distant VTE predictors, only BMI was identified as significant risk factor (Odds Ratio [OR] 1.12, 95% Confidence Interval [CI] 1.00, 1.23). When considering all VTE events together, BMI (Odds Ratio [OR] 1.13, 95% Confidence Interval [CI] 1.05, 1.22) and PLT count (Odds Ratio [OR] 1.13, 95% Confidence Interval [CI] 1.05, 1.22) were significant. Using Fluoropyrimidine chemotherapy increased the risk by 10-fold (Odds Ratio [OR] 10.06, 95% Confidence Interval [CI] 1.60, 63.05). No other significant predictors were found considering all VTE events. Figure 1 shows the summary of odds ratios of all potential predictive factors. For PICC line related DVT prediction, the exploratory logistic regression model was made on the items of Michigan score variables, and no significant predictors were found.

## Discussion

A common problem for cancer patients which could interrupt or delay cancer systemic treatment and affect the morbidity significantly is the PICC-related DVT. Our study found the symptomatic PICC related DVT incidence is 13%. Many retrospective design studies reported an incidence of symptomatic PICC-related thrombosis, ranging from 1% to 18 with few studies hit the limit of 28% [3, 4, 14–16, 19].However, there were several prospective studies reporting higher asymptomatic or incidental PICC -related DVT incidence, ranging from 27 to 71% [4, 14, 17, 20, 21].The possible causes for the wide variation in reported incidence may be related to study population, study design, the diagnosis method (Doppler US or venography), screening population (symptomatic or asymptomatic), the inconsistency in the definition of the VTE events (the difficulty in distinguishing mural thrombosis from central venous catheter occlusion by the catheter sleeve) and differences in the quality of patient surveillance. Implanted ports were associated with roughly 60% a relative risk reduction in cancer related thrombosis (OR = 0.43; 95% CI, 0.23–0.80) compared with PICC lines [13]. The possible explanation might be related to the relative less risk of endothelial injury because there is less movement of the catheter with port devices and the lower incidence of infection.

Physiologically speaking, this adverse event is not surprising. The PICC line is occupying much of the cross-sectional diameter of peripheral veins of the arm which predispose to venous stasis [22]. Peripherally inserted central catheter tips often displace and injure endothelium as they are prone to migration [23]. The infusion of chemotherapy is also carrying prothrombotic risk and with all above factors, PICC line often creating the perfect media for thrombosis [24]. Risk factors of catheter-associated thrombosis can be categorized to three types although they reported in different studies with great variation:

1. Chemotherapy type and use of prophylactic anticoagulant.
2. Patient’s factor like recent trauma or surgery, cancer, history of VTE, older age and renal failure
3. Catheter size, type, tip location, insertion site, numbers of venous insertions, and the catheter dwell time [25].

A recent study looked at PICC related thrombosis in all hospital patients found a number of significant risk factors for developing a PICC related DVT which included PICC size, previous DVT and surgery lasting more than one hour [10]. Another study identified that DVT risk correlated to catheter diameter, with DVT rates of 0,1and 6.6% per catheters sized < 3-F, 4-F and 5-F respectively [26]. The importance of lumen size was further highlighted in a follow-on study [27]. In our study the catheter size used was uniform of 4-F and all PICC insertions were conducted by interventional radiologist under fluoroscopy guidance. One study identified that the inappropriate choice of central venous access device and insertion technique as important risk factors for post-procedure complications, particularly in critically ill patients [28].However, one study that conducted on cancer patients with PICC line, none of the above-mentioned variables were predictive of PICC line related DVT and reported that co-morbidities such as diabetes, COPD and advanced cancer did predict DVT [3]. Lee et al. identified previous catheterization, more than one insertion attempt and ovarian cancer as being associated with an increased incidence of DVT [29].

In our study the median time required for PICC line related DVT development was 13 days 95% CI (12, 32). A large prospective study in the USA reported that 70% of thrombosis events occurred in the first week of insertion while 30% developed in the second week, after which no thromboses were identified [30]. These findings are similar to other studies conclusion in this aspect like one study which found PICC-related thrombosis forming at a mean-time of 12.4 ± 11 days while other studies also reported a mean thrombosis forming time of 15 days [16, 31, 32].Our finding is concords with previous literature reports and suggested the significance of prevention in the first 2 weeks after PICC insertion.

Significant risk factors in our study have been identified by multivariable logistic regression analyses which are smoking and high BMI with significant correlation to the PICC line related DVT while high PLT count and Fluropyrimidine containing chemotherapy found to have a significant relationship to all VTE events. In a large meta-analysis, involving 32 observational studies, found an increased risk of VTE with smoking independent of other risk factors [33]. Possible biological explanations for this relationship are procoagulant state, higher level of plasma fibrinogen and inflammation status which all may underlie the association between smoking and VTE risk [34–36]. Also, it has been shown that the fibrinogen concentration decreased quickly after smoking cessation [37, 38]. A high PLT count at the time of catheter insertion seems to be correlated with the rate of thrombotic complications in cancer patients which was demonstrated in one study that reported a lower risk of central venous catheter-related DVT in cancer patients with a low PLT count [39]. The influence of chemotherapy upon the rate of all VTE events in our cohort might relate to the increase risk for VTE in general with chemotherapy which is around more than two-fold [40]. In our study, 5-fluorouracil containing chemotherapy was associated with an increased risk of type VTE. This effect could be explained by the well-known pro-thrombotic and endothelial effects of 5-fluorouracil [41].

Compared with previous studies, our study had some advantage by exploring the distant VTE events during the period of PICC line insertion which showed a similar incidence of 23(13%) patients of the cohort; two of them had the PICC line related DVT already. The distribution of distant VTE was 18(78%) patients as PE while the lower limbs DVT recorded in only 5(22%) patients. The incidence of PE associated with the central venous catheter in cancer patients ranging from 15 to 25% [4].Two studies measured the incidence of symptomatic CTPA proven PE in cancer patients with the central venous catheter -related DVT and the incidence was 25 and 30%, respectively. However, both studies are of small size [42, 43].

The recorded symptoms in our study for patients who developed PICC line related DVT included swollen upper arm, forearm, axilla with discomfort or pain over the upper limb with inserted PICC line. Of note, some patients had veins engorgement over the affective side. In a meta-analysis, doppler ultrasound with compression was reported to have a pooled sensitivity of 91% and specificity of 93% in diagnosing upper extremity venous thrombosis [44].It is safe,low cost, non-invasive and fast making it the ideal method for diagnosis.

The current guidance does not recommend thromboprophylaxis in the setting of a long-term PICC line in cancer patients as no current evidence suggests that thromboprophylaxis is helpful to prevent these events despite a number of randomized controlled trials(RCT) [45, 46]. Moreover, a recent meta-analysis of 12 clinical trials assessing either primary prophylaxis dose heparins or low dose Warfarin in cancer patients with central venous catheters failed to show any benefit on the primary endpoints studied [47].

But there were still limitations in our study like determination of catheter tip position in those patients who developed the PICC line related DVT prior to its removal and assessing the vein lumen to catheter dimension ratio which considered as risk factors for PICC related DVT. The position of the tip at the junction of the superior vena cava and the right atrium may be a protective measure due to a greater dilutional effect when chemotherapeutic agents are infused or because the likelihood that the tip of the catheter will be in direct contact with the endothelium is a lower. Finally, due to the relatively small number of patients, we were unable to do internal validation For the Michigan score which is a PICC line related DVT prediction module.

## Conclusion

VTE is a common complication in medical oncology patients who underwent insertion PICC line insertion for chemotherapy. Risk of distant VTE is high as well as the PICC line related DVT and the risk of the PICC line related DVT is higher in the first 2 weeks after PICC insertion. We concluded that high BMI,high PLTs count and Fluropyrimidine containing chemotherapy are all significant risk factors for VTE events recorded while smoking and high BMI are significantly contributing to the high rate of the PICC line related DVT. There is no consensus on the role of prophylactic anticoagulation to reduce its incidence. Further study is required to generate risk prediction models to identified patients at higher risk for PICC line related DVT.

We recommend keeping a low threshold to arrange a Doppler ultrasound in any cancer patient with the PICC line with presentation such as pain and/or swelling in the same arm with PICC line, as the incidence is high.Further research on risk factors and the consequences of PICC thrombosis would also be very helpful. As our study identified some reversible risk factors that significantly associated with the PICC related DVT(smoking and high BMI), further patient education is advisable and probably prospective study after these risk factors modification will consolidate our finding. We recommend also further validation for the new Michigan Risk Score for PICC-Related Thrombosis which might be useful for estimation the risk of catheter-related thrombosis prior to inserting a PICC, support testing for thrombosis in patients with vague symptoms and can help about the anticoagulation period in patients with confirmed DVT related to PICC with a higher score. Finally, the consideration of Port-a-cath instead of PICC line in cancer patient with high risk of DVT is still not a standard option in the UK despite the lower risk of line related thrombosis and infection which worth further research.

## Abbreviations

BMI: Body Mass Index
CI: Confidence Interval
CRT: Clinical Randomised Trial
CT: Computed Tomography
CTPA: Computed Tomography Pulmonary Angiography
DVT: Deep Venous Thrombosis
OR: Odd Ratio
PE: Pulmonary Embolism
PICC: Peripheral Inserted Central Catheter
PLT: Platelet
VTE: Venous Thromboembolism
WBC: White Blood Cell

## References

[1] O'Brien J (2013). Insertion of PICCs with minimum number of lumens reduces complications and costs. J Am Coll Radiol.

[2] Gibson C (2013). Peripherally inserted central catheters: use at a tertiary care pediatric center. J Vasc Interv Radiol.

[3] Aw A (2012). Incidence and predictive factors of symptomatic thrombosis related to peripherally inserted central catheters in chemotherapy patients. Thromb Res.

[4] Verso M, Agnelli G (2003). Venous thromboembolism associated with long-term use of central venous catheters in cancer patients. J Clin Oncol.

[5] Chopra V (2012). The problem with peripherally inserted central catheters. JAMA.

[6] Chopra V (2013). Risk of venous thromboembolism associated with peripherally inserted central catheters: a systematic review and meta-analysis. Lancet.

[7] Lobo BL (2009). Risk of venous thromboembolism in hospitalized patients with peripherally inserted central catheters. J Hosp Med.

[8] Liem TK (2012). Peripherally inserted central catheter usage patterns and associated symptomatic upper extremity venous thrombosis. J Vasc Surg.

[9] Sperry BW, Roskos M, Oskoui R (2012). The effect of laterality on venous thromboembolism formation after peripherally inserted central catheter placement. J Vasc Access.

[10] Evans RS (2010). Risk of symptomatic DVT associated with peripherally inserted central catheters. Chest.

[11] Winters JP (2015). Central venous catheters and upper extremity deep vein thrombosis in medical inpatients: the medical inpatients and thrombosis (MITH) study. J Thromb Haemost.

[12] Fallouh N (2015). Peripherally inserted central catheter-associated deep vein thrombosis: a narrative review. Am J Med.

[13] Saber W (2011). Risk factors for catheter-related thrombosis (CRT) in cancer patients: a patient-level data (IPD) meta-analysis of clinical trials and prospective studies. J Thromb Haemost.

[14] Chemaly RF (2002). Venous thrombosis associated with peripherally inserted central catheters: a retrospective analysis of the Cleveland Clinic experience. Clin Infect Dis.

[15] Cortelezzia A (2003). Central venous catheter-related complications in patients with hematological malignancies: a retrospective analysis of risk factors and prophylactic measures. Leuk Lymphoma.

[16] King MM (2006). Peripherally inserted central venous catheter-associated thrombosis: retrospective analysis of clinical risk factors in adult patients. South Med J.

[17] Paauw JD (2008). The incidence of PICC line-associated thrombosis with and without the use of prophylactic anticoagulants. JPEN J Parenter Enteral Nutr.

[18] Itkin M (2014). Peripherally inserted central catheter thrombosis – reverse tapered versus nontapered catheters: a randomized controlled study. J Vasc Interv Radiol.

[19] Loughran SC (1995). Peripherally inserted central catheters: a report of 2,506 catheter days. JPEN J Parenter Enteral Nutr.

[20] Kamphuisen PW,et al (2012). Catheter-related thrombosis: lifeline or a pain in the neck?. Hematology Am Soc Haematol Educ Program.

[21] Liu Y, et al. Peripherally inserted central catheter thrombosis incidence and risk factors in cancer patients: a double-center prospective investigation 2015;11:153–160. 10.2147/TCRM.S73379 PMC4321638 25673995

[22] Nifong TP, McDevitt TJ (2011). The effect of catheter to vein ratio on blood flow rates in a simulated model of peripherally inserted central venous catheters. Chest.

[23] Song L (2014). Malposition of peripherally inserted central catheter: experience from 3012 cancer patients. Int J Nurs Pract.

[24] Ackerknecht EH (1953). Rudolf Virchow. Doctor, statesman, anthropologist.

[25] Gallieni M (2008). Vascular access in oncology patients. CA Cancer J Clin.

[26] Grove JR (2000). Venous thrombosis related to peripherally inserted central catheters. J Vasc Interv Radiol.

[27] Evans RS (2013). Reduction of peripherally inserted central catheter-associated DVT. Chest.

[28] Cotogni P (2014). Focus on peripherally inserted central catheters in critically ill patients. World J Crit Care Med.

[29] Lee AY (2006). Incidence, risk factors, and outcomes of catheter-related thrombosis in adult patients with cancer. J Clin Oncol.

[30] Walshe LJ (2002). Complication rates among cancer patients with peripherally inserted central catheters. J Clin Oncol.

[31] Ng PK (1997). Peripherally inserted central catheters in general medicine. Mayo Clin Proc.

[32] Ong B (2006). Peripherally inserted central catheters and upper extremity deep vein thrombosis. Australas Radiol.

[33] Cheng YJ, Liu ZH, Yao FJ (2013). Current and former smoking and risk for venous thromboembolism: a systematic review and meta-analysis. PLoS Med.

[34] Lee KW, Lip GYH (2003). Effects of lifestyle on hemostasis, fibrinolysis, and platelet reactivity: a systematic review. Arch Intern Med.

[35] Yarnell JW, Sweetnam PM, Rumley A, Lowe GD (2000). Lifestyle and hemostatic risk factors for ischemic heart disease: the Caerphilly study. Arterioscler Thromb Vasc Biol.

[36] Oger E, Lacut K, Van Dreden P (2003). High plasma concentration of factor VIII coagulant is also a risk factor for venous thromboembolism in the elderly. Haematologica.

[37] Feher MD, Rampling MW, Brown J (1990). Acute changes in atherogenic and thrombogenic factors with cessation of smoking. J R Soc Med.

[38] Bakhru A, Erlinger TP (2005). Smoking cessation and cardiovascular disease risk factors: results from the third National Health and nutrition examination survey. PLoS Med.

[39] Haire WD (1990). Hickman catheter-induced thoracic vein thrombosis: frequency and long-term sequelae in patients receiving high-dose chemotherapy and marrow transplantation. Cancer.

[40] Blom JW (2006). Incidence of venous thrombosis in a large cohort of 66,329 cancer patients: results of a record linkage study. J Thromb Haemost.

[41] Polk A (2014). A systematic review of the pathophysiology of 5-fluorouracil-induced cardiotoxicity. BMC Pharmacol Toxicol.

[42] Manuel Monreal. Upper-extremity deep venous thrombosis and pulmonary embolism. A prospective study Chest 1991; 99(2). 10.1378/chest.99.2.280 1989783

[43] Monreal M. Pulmonary embolism in patients with upper extremity DVT associated to venous central lines--a prospective study. Thromb Haemost. 1994;72(4). 7878630

[44] Chin EE (2005). Sonographic evaluation of upper extremity deep venous thrombosis. J Ultrasound Med.

[45] Karthaus M (2006). Dalteparin for prevention of catheter-related complications in cancer patients with central venous catheters: final results of a double-blind, placebo-controlled phase III trial. Ann Oncol.

[46] Young AM (2009). Warfarin thromboprophylaxis in cancer patients with central venous catheters (WARP): an open-label randomised trial. Lancet.

[47] Akl EA (2014). Anticoagulation for people with cancer and central venous catheters. Cochrane Database Syst Rev.

